# The effect of e-cigarettes on smoking cessation and cigarette smoking initiation: An evidence-based rapid review and meta-analysis

**DOI:** 10.18332/tid/131624

**Published:** 2021-01-13

**Authors:** Ying-Ying Zhang, Fan-Long Bu, Fei Dong, Jian-Hua Wang, Si-Jia Zhu, Xiao-Wen Zhang, Nicola Robinson, Jian-Ping Liu

**Affiliations:** 1 Centre for Evidence-Based Chinese Medicine, Beijing University of Chinese Medicine, Beijing, China; 2 Beijing Children's Hospital, Capital Medical University, Beijing, China; 3 Science and Technology Department, Liaoning University of Traditional Chinese Medicine, Shenyang, China; 4 School of Health and Social Care, London South Bank University, London, United Kingdom

**Keywords:** e-cigarettes, smoking cessation, smoking initiation, rapid review, systematic review

## Abstract

**INTRODUCTION:**

The contribution made by e-cigarettes to smoking cessation continues to be controversial. Reports suggest that teenagers are becoming increasingly addicted to e-cigarettes and that e-cigarette use in adolescents is associated with subsequent cigarette smoking.

**METHODS:**

Systematic searches of eleven databases were conducted (January 2015 to June 2020). Systematic reviews, randomized controlled trials (RCTs) and cohort studies comparing e-cigarettes with placebo e-cigarettes, nicotine replacement therapy (NRT) or no e-cigarette use were included. The two primary outcomes were smoking cessation among smokers and smoking initiation among non-smoking teenagers. The secondary outcome was adverse events. Data were synthesized using risk ratio (RR) or adjusted odds ratio (AOR) with 95% confidence interval (CI).

**RESULTS:**

Six systematic reviews, 5 RCTs and 24 cohort studies were identified. For smoking cessation, findings from 4 systematic reviews indicated that e-cigarettes contributed to cessation while one found the opposite. Meta-analysis of 5 RCTs suggested that e-cigarettes were superior to NRT or placebo for smoking cessation (RR=1.55; 95% CI: 1.00–2.40; I^2^=57.6%; low certainty; 5 trials, n=4025). Evidence from 9 cohort studies showed that e-cigarette use was not associated with cessation (AOR=1.16; 95% CI: 0.88–1.54; I^2^=69.0%; n=22220). Subgroup analysis suggested that intensive e-cigarette use may be associated with cessation. In terms of smoking initiation, adolescents who ever used e-cigarettes had a greater risk for smoking initiation than non-users (AOR=2.91; 95% CI: 2.61–3.23; I^2^=61.0%; 15 trials, n=68943), the findings were consistent with one included systematic review. No serious adverse events were reported in the included studies.

**CONCLUSIONS:**

Low certainty evidence suggests that e-cigarettes appear to be potentially effective for smoking cessation. The use of e-cigarettes in adolescents may be associated with smoking initiation. No serious adverse events were reported.

## INTRODUCTION

Electronic cigarettes, also called e-cigarettes are electronic nicotine delivery systems (ENDS). They can deliver vaporized liquid mainly containing nicotine and propylene glycol through the mouth into the lungs, mimicking the effects of conventional cigarette smoking^1^. Reportedly, the e-cigarette was invented by the Chinese pharmacist Hon Lik in 2003, and internationally patented in 2007^2^. E-cigarettes have been commercially available in Europe and America since 2006 as a cigarette substitute^3^. E-cigarettes have always been advertised as healthy and popular smoking cessation tools^4^. Reports have suggested that achieving abstinence by using e-cigarettes was comparable to nicotine replacement therapy (NRT), and e-cigarettes also appear to effectively decrease cigarette consumption^5,6^. This has resulted in a dramatic increase in the use of e-cigarettes worldwide. The estimated global value of e-cigarette sales was up to US$3.5 billion in 2015^7^. A national large-scale survey from 28 EU member states reported that there were about 48.5 million e-cigarette users in 2016^8^.

Increasingly teenagers are becoming addicted to e-cigarettes. Between 2016 and 2017, the proportion of e-cigarette users among those aged 11 to 16 years increased from 7% to 11%, respectively, across the UK^9^. The proportion of US high school teenagers who had tried e-cigarettes in the last month rose from 1.5% to 16.0% between 2011 and 2015, a more than 10-fold increase^10^. In 2019, it was estimated that among high school students in the US, 27.5% (95% CI: 25.3–29.7) were current e-cigarette users^11^. Worse still, many studies appear to report that use of e-cigarettes by young people increases the risk of subsequent cigarette smoking^12^, and that minors are an emerging new smoking population^13^.

Concerns have been expressed about the safety of e-cigarettes. The US Food and Drug Administration (FDA) reported that e-cigarettes contained some toxic heavy metals and the concentration of heavy metals released by e-cigarettes was much higher than conventional cigarettes^14^. These were associated with the development of cancer^15^ and the occurrence of coronary events^16^. In light of the above, the FDA banned flavored e-cigarette sales in January 2020, as flavored e-cigarettes had the most appeal to teenagers^17^. On 1 November 2019, China as the birthplace of e-cigarettes announced that the online purchase of e-cigarettes would be completely banned in China^18^. Previously, two randomized controlled trials (RCTs)^5,6^ indicated that e-cigarettes were potentially effective in smoking cessation among adult smokers. Published studies on cigarette smoking initiation associated with e-cigarette use among adolescents have been primary studies and there has been limited evidence synthesis. There are few evidence-based studies that have comprehensively evaluated the benefits and risks of e-cigarettes. Therefore, a rapid review was conducted to evaluate the effects of e-cigarettes on smoking cessation among smokers and the risks for smoking initiation among non-smoking adolescents, and their safety.

## METHODS

Due to the urgent need to inform ongoing policy, a rapid review methodology was employed^19^. This type of simplified systematic review is helpful in providing a timely synthesis for decision makers. Streamlined methods are usually used in a rapid review, which usually includes limiting retrieval dates and databases^19^. This rapid review and meta-analysis are reported following Preferred Reporting Items for Systematic Reviews and Meta-Analyses (PRISMA)^20^ statement. A PRISMA statement with a checklist of items that should be included in reports of systematic reviews is included in the Supplementary file.

### Eligibility criteria

Systematic reviews based on RCTs or comparative observational studies (cohort and cross-sectional studies), parallel group RCTs and controlled cohort studies were included. The study population comprised adult smokers and non-smoking adolescents, who had no serious diseases or pregnancy. The study interventions were e-cigarettes. The controls referred to placebo e-cigarettes (without nicotine), NRT or no treatment. The primary outcomes were smoking cessation among smokers and smoking initiation among adolescents. The secondary outcome was occurrence of adverse events. Experimental studies and studies that failed to report the minimal required information were excluded. There was no need for approval from an ethics committee or agreement of participants in this study as data were extracted from publications that were in the public domain.

### Search strategy

Databases searched included PubMed, Web of Science, MEDLINE, EMBASE, the Cochrane library, China National Knowledge Infrastructure (CNKI), Chinese Scientific Journal Database (VIP), SinoMed and Wanfang, from January 2015 to June 2020. The reference lists included in the systematic reviews were also searched. A systematic search was also conducted in Clinical Trials.gov (www.clinicaltrials.gov) and Chinese Clinical Trial Registry (http://www.chictr.org.cn/index.aspx). Search terms included: e-cigarettes, electronic cigarettes, electronic nicotine delivery systems, vape, and vaping. An example of the PubMed search strategy is given in the Supplementary file.

### Study selection and data extraction

After removing duplicates, two authors (SJZ and FLB) independently screened studies by title and abstract, according to the eligibility criteria. In the full text screening process, uncertainty and insufficient information was determined for eligibility through full texts. Disagreements were resolved by discussion or arbitrated by a methodologist (JPL). Reasons for excluding studies were recorded at this stage. After identification of studies, data were extracted independently by two authors (SJZ and FLB) and included: characteristics of study design (study type, setting, and funding); details of PICO (participants, interventions, comparisons, and outcomes); and follow-up and adjusted factors. Any disagreements were resolved by discussion or third-party adjudication.

### Quality assessments

The methodological quality assessment of each included study was performed independently by two review authors (FLB and SJZ). Cochrane Risk of Bias tool (ROB)^21^ was employed to assess whether the risk of bias of RCTs was low, high or unclear according to its seven domains. A Measurement Tool to Assess Systematic Reviews (AMSTAR-2)^22^ which has 16 items with 7 key items was employed to assess the quality of the included systematic reviews; the overall confidence for each review was assessed as high, moderate, low or critically low. The quality of cohort studies was assessed by Newcastle-Ottawa Scale (NOS)^23^, which is composed of 8 items and includes three subscales: selection of studies, comparability of studies, and the ascertainment of exposure. The maximum score is 9 points and any study scoring >5 points was considered of moderate to high quality^23^. Grading of Recommendations Assessment, Development and Evaluation (GRADE)^24^ was employed to assess the certainty of evidence from RCTs in five domains (risk of bias, directness, precision, consistencies, and publication bias).

### Data synthesis

Qualitative and quantitative methods were adopted to synthesize the findings. The findings from systematic reviews were narratively described. Data are presented as risk ratios (RRs) or adjusted odds ratio (AORs) with 95% confidence intervals (CIs). Meta-analysis was performed by Stata version 14.0 software when the trials had acceptable heterogeneity and similarities in clinical characteristics. The random effects model (REM) was utilized in the meta-analysis to consider potential sources of clinical heterogeneity. The I^2^ statistic was employed to assess statistical heterogeneity^21^. When p<0.10 and I^2^>50%, the heterogeneity may be considered as high^21,25^. To explain heterogeneity, we predefined the subgroup analysis by the frequency of e-cigarette use: intensive use (daily or regular use for at least one month) versus intermittent or irregular use. Sensitivity analysis was employed to test the robustness of the results when the primary outcomes were statistically different for the following three methodological domains: reported clear randomization concealment or not; placebo used or not; and reported lost to follow-up or not. Funnel plots were employed to detect the possibility of publication bias if ≥10 studies were included in a meta-analysis.

## RESULTS

### Screening

**Figure 1:**
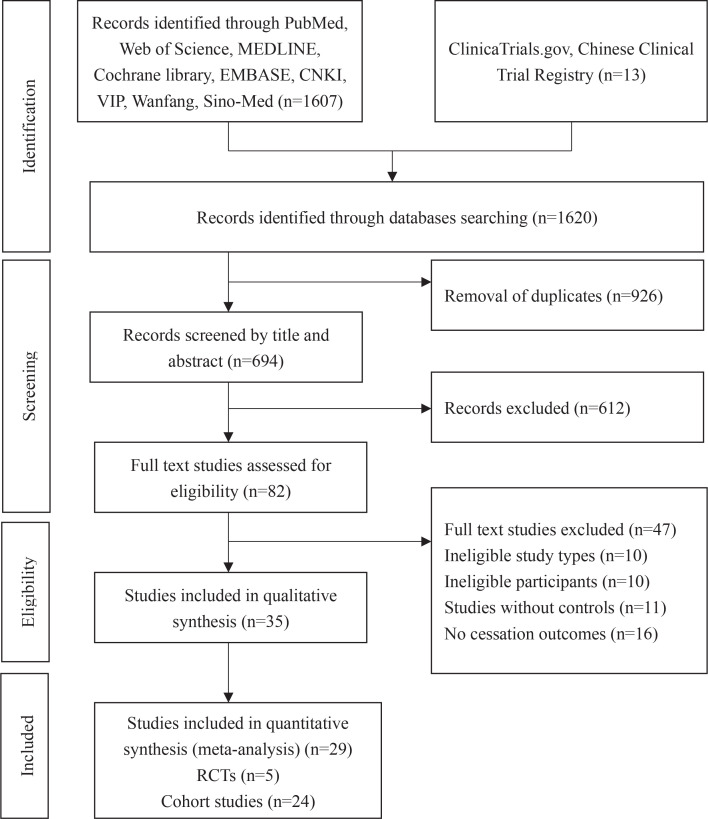
Flow chart of study selection

Initially, 1620 records were retrieved and 926 duplicates were removed. In all, 612 records were excluded by scanning the title and abstract due to irrelevant studies, protocols, clinical studies, case-control studies or cross-sectional surveys, commentary of included studies, and studies that provided only abstracts. This left 82 remaining records, of these, 47 studies were excluded through full-text screening due to ineligible study design, uncontrolled cohort studies or lack of cessation outcomes. Finally, 35 studies were included after full-text screening. Of these studies, findings from 6 systematic reviews^26-31^ were narratively described. The screening process is shown in Figure 1.

### Description of studies

The characteristics of the included studies are shown in Tables 1 and 2. Included in the 35 studies identified were: 6 systematic reviews^26-31^, 5 RCTs^32-36^, and 24 cohort studies^37-60^. These studies were classified into two categories based on the two primary outcome measures. Nineteen studies^26-30,32-45^ examined the effects of e-cigarette use on smoking cessation, and the remaining 16 studies^31,46-60^ explored the causality between e-cigarette use and smoking initiation among teenagers. The study population included adult smokers and non-smoking adolescents and young adults. The definition of e-cigarette use was ‘current use’, ‘ever use’ or ‘past 30-day use’. In terms of the included 6 systematic reviews, 5 reviews^26-30^ focused on smoking cessation among adult smokers, and included either RCTs, cohort studies or cross-sectional studies. Another systematic review^31^ was based on 9 cohort studies and explored the association between e-cigarette use and subsequent smoking initiation. The 5 RCTs^32-36^ involved data from 4025 adult smokers. The sample size ranged from 99 to 886. Three trials^32,^ ^34,35^ compared the effect of e-cigarettes with NRT on smoking cessation. The other 2 RCTs^33,36^ compared the effect of nicotine e-cigarettes with placebo or no treatment. A total of 24 cohort studies were identified. The sample size ranged from 146 to 17318 with a total of 91985 participants. Nine studies^37-45^ focused on smoking cessation, of which, 4 studies^37-39,42^ overlapped with the included systematic reviews^26-30^. Another 15 cohort studies^46-60^ focused on the association between e-cigarette use and subsequent smoking initiation among teenagers. Of these, six^46,49-51,54,56^ studies overlapped with the included systematic review^31^. The follow-up time varied from 6 months to 2 years.

**Table 1:** Characteristics of the included systematic reviews and randomized controlled trials

*Study and Year*	*Location*	*Population/intervention*	*Comparison*	*Outcomes*	*Included study types*
Kalkhoran et al.^26^ (2016)	USA	Adult smokers with nicotine EC use	Non-EC users	Probability of smoking cessation	15 cohort studies, 3 cross-sectional studies and 2 clinical trials
El Dib et al.^27^ (2017)	Brazil	Adult smokers with nicotine EC use	Placebo EC	Probability of smoking cessation	3 RCTs and 9 prospective cohort studies
Hartmann-Boyce et al.^28^ (2016)	UK	Adult smokers with nicotine EC use	Placebo EC	Probability of smoking cessation	3 RCTs and 21 cohort studies
Khoudigian et al.^29^ (2016)	Canada	Adult smokers with nicotine EC use	Placebo EC	Probability of smoking cessation	3 RCTs and 2 comparative observational studies
Rahman et al.^30^ (2015)	Australia	Adult smokers with nicotine EC use	Placebo EC	Probability of smoking cessation	2 RCTs, 2 cohort studies and 2 cross-sectional studies
Soneji et al.^31^ (2017)	Lebanon	Non-smoking teenagers with current or past 30-day nicotine EC use	Never or non-past 30-day EC users	Probability of cigarette smoking initiation	9 cohort studies
Hajek et al.^32^ (2019)	UK	Nicotine EC users (n=438)	NRT (n=446)	1-year abstinence rate	RCT
Tseng et al.^33^ (2016)	USA	4.5% nicotine EC users (n=50)	Placebo EC (n=49)	Abstinence rate at week 3	RCT
Lee et al.^34^ (2019)	Korea	Nicotine EC users (n=75)	Nicotine gum (n=75)	Abstinence rate at 9–24 weeks	RCT
Li et al.^35^ (2019)	UK	Nicotine EC users (n=439)	NRT (n=447)	1-year abstinence rate	RCT
Halpern et al.^36^ (2018)	USA	Nicotine EC users (n=1199)	No treatment (n=813)	6-month abstinence rate	RCT

EC: electronic cigarette. NRT: nicotine replacement therapy.

**Table 2:** Characteristics of the included cohort studies

*Study and Year*	*Location*	*Exposure*	*Population*	*Comparison*	*Follow-up*	*Outcomes*	*Adjusted factors*	*Lost to follow-up (%)*
follow-upShi et al.^37^ (2016)	USA	Ever nicotine EC users (n=82)	2454 current adult smokers	Non-EC users (n=936)	1 year	Probability of smoking cessation	Demographics and baseline cigarette dependence level	53.3
Biener et al.^38^ (2015)	USA	Ever nicotine EC users, intensive (n=111), intermittent (n=220)	695 smokers were categorized as intensive, intermittent and non-EC users	Non-EC users (n=364)	2 years	Probability of smoking cessation	Demographics and tobacco dependence	49.4
Manzoli et al.^39^ (2015)	Italy	Ever nicotine EC users (n=223)	1355 adult (aged 30–75 years) smokers	Non-EC users (n=480)	2 years	Probability of smoking cessation	Sociodemographic factors alcohol use and years of smoking	31.2
Zhuang et al.^40^ (2016)	USA	Ever nicotine EC users: long-term (n=72) short-term (n=456)	2028 smokers were categorized as long-term and short-term EC users	Non-EC users (n=1500)	2 years	Probability of smoking cessation	Sociodemographics, smoking status and intention to cessation	32.6
Pasquereau et al.^41^ (2017)	France	Ever nicotine EC users (n=252)	2057 smokers aged 15–85 years	Non-EC users (n=1805).	6 months	Probability of smoking cessation	Socioeconomic variables and smoking behaviors	31.4
Al-Delaimy et al.^42^ (2015)	USA	Ever nicotine EC users (n=236)	1000 adult smokers	Non-EC users (n=177)	1 year	Probability of smoking cessation	Sociodemographic characteristics	60.0
Kalkhoran et al.^43^ (2019)	USA	Daily nicotine EC users (n=299), non-daily EC users (n=1523)	8218 adult smokers were categorized as daily and non-daily EC users	Non-EC users (n=6379)	1 year	Probability of smoking cessation	Sociodemographic characteristics and smoking status	2.0
Jackson et al.^44^ (2019)	UK	Nicotine EC users (n=292)	1709 adult smokers (aged ≥16 years)	Non-EC users (n=1089)	1 year	Probability of smoking cessation	Sociodemographics, smoking status, motivation and quit attempts	9.8
Kalkhoran et al.^45^ (2019)	USA	Nicotine EC users (n=762)	4948 adult smokers	Non-EC users (n=4156)	1 year	Probability of smoking cessation	Sociodemographics, smoking status, education, region, and nicotine dependence	<4.0
Wills et al.^46^ (2016)	USA	Nicotine EC users (n=215)	2338 non-smoking students (mean age 14.7 years)	Non-EC users (n=926)	1 year	Probability of smoking initiation	Age, ethnicity and rebelliousness		4.20
Aleyan et al.^47^ (2017)	Canada	Non-susceptible current nicotine EC users (n=73)	9501 grade 9–11 non-smoking students	Non-susceptible non-current EC users (n=6616)	2 years	Probability of smoking initiation	Gender, grade, ethnicity and social risk factors	21.1
Goldenson et al.^48^ (2017)	USA	Low nicotine EC users (n=52), medium (n=35), high users (n=21)	3252 grade 10–11 non-smoking students	Placebo-EC users (n=73)	6 months	Probability of smoking initiation	Interpersonal, intrapersonal and demographic factors	5.0
Leventhal et al.^49^ (2015)	USA	Ever nicotine EC users (n=222)	2530 non-smoking teenagers (mean age=14.1 years)	Non-EC users (n=2308)	6 months 12 months	Probability of smoking initiation	Sociodemographic, environmental and intrapersonal risk factors	1.1
Primack et al.^50^ (2015)	USA	Ever nicotine EC users (n=16)	694 non-smokers aged 16–26 years, attitudinally non-susceptible to smoking	Non-EC users (n=628)	1 year	Probability of smoking initiation	Sociodemographic factors and maternal educational level	30.3
Barrington-Trimis et al.51 (2016)	USA	Nicotine EC users (n=146)	146 non-smokers (mean age 17.4 years)	Frequency matched non-EC users (n=152)	16 months	Probability of smoking initiation	Gender, ethnicity and grade	30.0
Barrington-Trimis et al.^52^ (2018)	USA	Nicotine EC users (n=673)	6258 non-smoking adolescents	Non-EC users (n=3891)	2 years	Probability of smoking initiation	Gender, race/ethnicity, grade, cohort	1.8
Primack et al.^53^ (2018)	USA	Ever nicotine EC users (n=16)	1506 non-smokers aged 18–30 years	Non-EC users (n=899)	18 months	Probability of smoking initiation	Sociodemographic, personal, and environmental covariates	39.2
Unger et al.^54^ (2016)	USA	Ever nicotine EC users (n=42)	1056 past 30-day non-smokers (mean age 22.7 years)	Non-EC users (n=1014)	1 year	Probability of smoking initiation	Age, sex, and past-month use of alcohol and other tobacco products	7.8
Hammond et al.^55^ (2017)	Canada	Past 30-day nicotine EC users (n=487)	17318 non-smoking adolescents and young adults	Non past-30 EC users (n=16831)	1 year	Probability of smoking initiation	Sociodemographic and smoking status	9.5
Spindle et al.^56^ (2017)	USA	Ever nicotine EC users (n=153)	Non-smoking youths (n=2316)	Non-EC users (n=2163)	1 year	Probability of smoking initiation	Anxiety, depression peer deviance and smoking status	17.8
Miech et al.^57^ (2017)	USA	Ever nicotine EC users	Non-smoking youths (n=246)	Non-EC users	1 year	Probability of smoking initiation	Sociodemographic characteristics and parental education	57.8
Chien et al.^58^ (2019)	Taiwan	Ever nicotine EC users (n=661)	12954 non-smoking students	Non-EC users (n=879)	2 years	Probability of smoking initiation	Sociodemographic characteristics and smoking status	10.1
Berry et al.^59^ (2019)	USA	Ever nicotine EC users (n=527)	Non-smoking youths (n=6123)	Non-EC users (n=5290)	2 years	Probability of smoking initiation	Sociodemographic characteristics, smoking status and behaviors	5.0
Mcmillen et al.^60^ (2019)	USA	Ever nicotine EC users (n=195)	Non-smoking youths (n=5776)	Non-EC users (n=5473)	1 year	Probability of smoking initiation	Demographic variables and psychosocial predictors	1.9

EC: electronic cigarette. NRT: nicotine replacement therapy.

### Quality assessment

**Figure 2:**
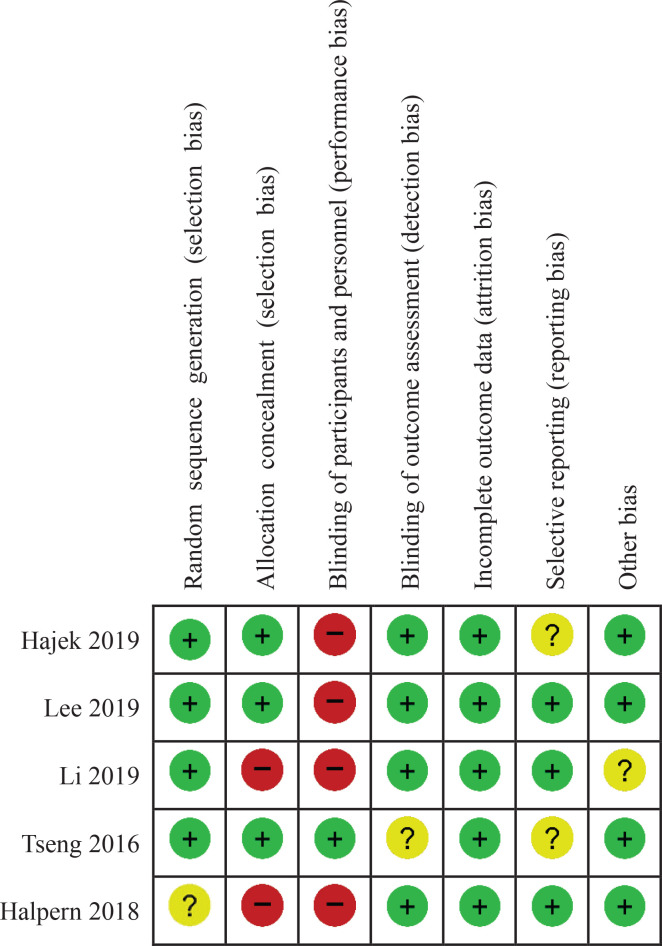
Risk of bias summary

There were no^26^ or one^28^ non-critical weakness in two systematic reviews, and these were rated as high confidence according to AMSTAR-2. Three^29-31^ were rated as low confidence due to the lack of a protocol and inadequate details of the included studies. One^27^ was of critically low confidence as it lacked a protocol and the lists of excluded studies. Details are shown in Table 3. In terms of the RCTs, the random sequence generation and allocation concealment for 3 RCTs^32-34^ were well described and were judged as having a low risk of bias. Blinding of the participants and personnel was only reported for one RCT^33^ and was rated as ‘low’ in the blinding domain. Apart from this, the risk of performance bias was rated as ‘high’ in the other 4 RCTs^32,34-36^, but the outcome assessment was blinded in these 4 RCTs and were rated as having low risk of detection bias. Intention-to-treat analysis was conducted in 5 RCTs^32-36^, and the attrition rate was <10%; all RCTs were assessed as having low risk of attrition bias. In terms of reporting bias, 3 RCTs^34-36^ were assessed as ‘low’ since the protocols were registered and the consistency between the outcomes described in protocol and actual outcomes in the results; the remaining 2 RCTs^32,33^ were assessed as ‘unclear’ due to lack of a registered protocol. The baseline data were comparable for 4 RCTs^32,34-36^, one^35^ was rated as ‘unclear’ due to the lack of baseline data. The risk of bias summary is shown in Figure 2. The quality of 24 cohort studies scored between 5 and 7 according to the NOS grading due to the higher number of dropouts and self-reported outcomes. Overall, the quality was considered to be satisfactory. Details are shown in Table 4.

**Table 3:** AMSTAR-2 assessment of the included systematic reviews

*Study and Year*	*AMSTAR-2 Items*																*Overall quality*
	*1*	*2*	*3*	*4*	*5*	*6*	*7*	*8*	*9*	*10*	*11*	*12*	*13*	*14*	*15*	*16*	
Kalkhoran et al.^26^ (2016)	Y	Y	Y	Y	Y	Y	Y	PY	Y	Y	Y	Y	Y	Y	Y	Y	high
El Dib et al.^27^ (2017)	Y	N	Y	PY	Y	Y	N	Y	Y	Y	PY	Y	Y	Y	Y	Y	critically low
Hartmann-Boyce et al.^28^ (2016)	Y	Y	Y	Y	Y	Y	Y	PY	Y	N	Y	Y	Y	Y	Y	Y	high
Khoudigian et al.^29^ (2016)	Y	N	Y	Y	Y	Y	Y	PY	Y	Y	Y	Y	Y	Y	Y	Y	low
Rahman et al.^30^ (2015)	Y	N	Y	Y	Y	Y	Y	N	Y	Y	Y	Y	Y	Y	Y	Y	low
Soneji et al.^31^ (2017)	Y	N	Y	Y	Y	Y	Y	Y	Y	N	Y	Y	Y	Y	Y	Y	low

Y: yes. N: no. PY: partial Y. CL: critically low. L: low. H: high. Item 1: Did the research questions and inclusion criteria for the review include the components of PICO?; Item 2: Did the report of the review contain an explicit statement that the review methods were established prior to the conduct of the review and did the report justify any significant deviations from the protocol?; Item 3: Did the review authors explain their selection of the study designs for inclusion in the review?; Item 4: Did the review authors use a comprehensive literature search strategy?; Item 5: Did the review authors perform study selection in duplicate?; Item 6: Did the review authors perform data extraction in duplicate?; Item 7: Did the review authors provide a list of excluded studies and justify the exclusions?; Item 8: Did the review authors describe the included studies in adequate detail?; Item 9: Did the review authors use a satisfactory technique for assessing the risk of bias (ROB) in individual studies that were included in the review?; Item 10: Did the review authors report on the sources of funding for the studies included in the review?; Item 11: If meta-analysis was performed, did the review authors use appropriate methods for statistical combination of results?; Item 12: If meta-analysis was performed, did the review authors assess the potential impact of ROB in individual studies on the results of the meta-analysis or other evidence synthesis?; Item 13: Did the review authors account for ROB in individual studies when interpreting/discussing the results of the review?; Item 14: Did the review authors provide a satisfactory explanation for, and discussion of, any heterogeneity observed in the result of the review?; Item 15: If they performed quantitative synthesis, did the review authors carry out an adequate investigation of publication bias (small study bias) and discuss its likely impact on the results of the review?; Item 16: Did the review authors report any potential sources of conflicts of interest, including any funding they received for conducting the review?

**Table 4:** Newcastle-Ottawa Scale assessment of the included cohort studies

*Study and Year*	*Newcastle-Ottawa Scale*			
	*Selection*	*Comparability*	*Outcome*	*Total score*
Shi et al.^37^ (2016)	3	2	1	6
Biener et al.^38^ (2015)	3	2	1	6
Manzoli et al.^39^ (2015)	3	2	1	6
Zhuang et al.^40^ (2016)	2	2	1	5
Pasquereau et al.^41^ (2017)	2	2	1	5
Al-Delaimy et al.^42^ (2015)	3	2	1	6
Kalkhoran et al.^43^ (2019)	2	2	1	5
Jackson et al.^44^ (2019)	2	2	2	6
Kalkhoran et al.^45^ (2019)	2	2	1	5
Wills et al.^46^ (2016)	2	2	2	6
Aleyan et al.^47^ (2017)	2	2	3	7
Goldenson et al.^48^ (2017)	2	2	2	6
Leventhal et al. ^49^ (2015)	2	2	2	6
Primack et al.^50^ (2015)	3	2	1	6
Barrington-Trimis et al.^51^ (2016)	2	2	1	5
Barrington-Trimis et al.^52^ (2018)	1	2	2	5
Primack et al.^53^ (2018)	2	2	1	5
Unger et al.^54^ (2016)	2	2	2	6
Hammond et al.^55^ (2017)	2	2	2	6
Spindle et al.^56^ (2017)	2	2	2	6
Miech et al.^57^ (2017)	2	2	1	5
Chien et al.^58^ (2019)	2	2	2	6
Berry et al.^59^ (2019)	2	2	1	5
Mcmillen et al.^60^ (2019)	2	2	1	5

### Effects of interventions

#### Smoking cessation

##### Findings from 5 systematic reviews

A Cochrane systematic review^28^ compared e-cigarettes with placebo e-cigarettes on smoking cessation, but only 2 RCTs^5,6^ were eligible for the meta-analysis. The abstinence rates for at least 6 months were 4% (placebo group) and 9% (e-cigarette group), suggesting that e-cigarettes were more likely to favor cessation (RR=2.29; 95% CI: 1.05–4.96; low certainty; 2 trials, n=662). This was consistent with 3 systematic reviews^27,29,30^. Another systematic review^26^ reported that the odds of cessation were 28% lower in the e-cigarette group than the non-use group (OR=0.72; 95% CI: 0.57–0.91; 20 trials, n=355011).

##### Findings from 5 RCTs

**Figure 3:**
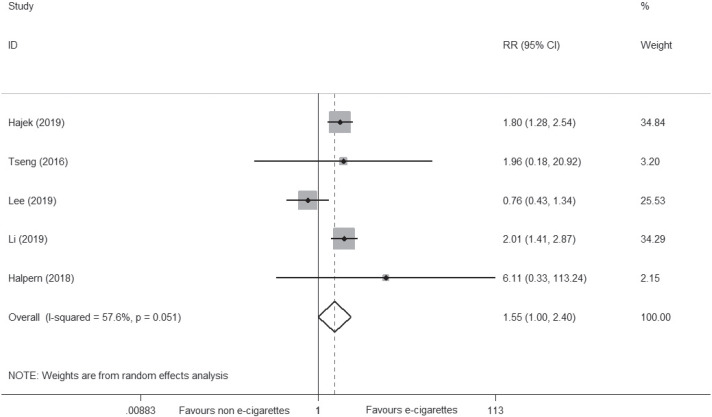
Meta-analysis of RCTs comparing e-cigarettes with NRT or placebo on smoking cessation

Five newly conducted RCTs^32-36^ reported the abstinence rate, with 180 of 2201 (8.2%) smokers achieving cessation in the e-cigarette group versus 103 of 1824 (5.6%) in the control group, suggesting that e-cigarettes may be superior to NRT or placebo on smoking cessation (RR=1.55; 95% CI: 1.00–2.40; I^2^=57.6%; low certainty; 5 trials, n=4025) (Figure 3, Table 5). However, the certainty of evidence was downgraded under GRADE assessment due to the small number of events (<300) and the inconsistency level (I^2^=57.6%). Details are shown in Table 5.

**Table 5:** Evidence summary of smoking cessation: e-cigarettes versus NRT or placebo

*Certainty assessment*							*Number of patients*		*Effect*		*Certainty*	*Importance*
No. of studies	Study design	Risk of bias	Inconsistency	Indirectness	Imprecision	Other considerations	EC	NRT or placebo	Relative (95% CI)	Absolute (95% CI)		
*Abstinence rate*												
5	Randomized trials	Not serious	Serious^a^	Not serious	Serious^b^	Undetected	180/2201 (8.2%)	103/1824 (5.6%)	RR=1.55 (1.00–2.40)	31 more per 1000 (from 0 fewer to 79 more)	⊕ ⊕ ◯ ◯ Low	Critical

a I2=57.6%.

b Number of events <300. EC: electronic cigarette. NRT: nicotine replacement therapy. CI: confidence interval. RR: risk ratio.

##### Findings from 9 cohort studies

**Figure 4:**
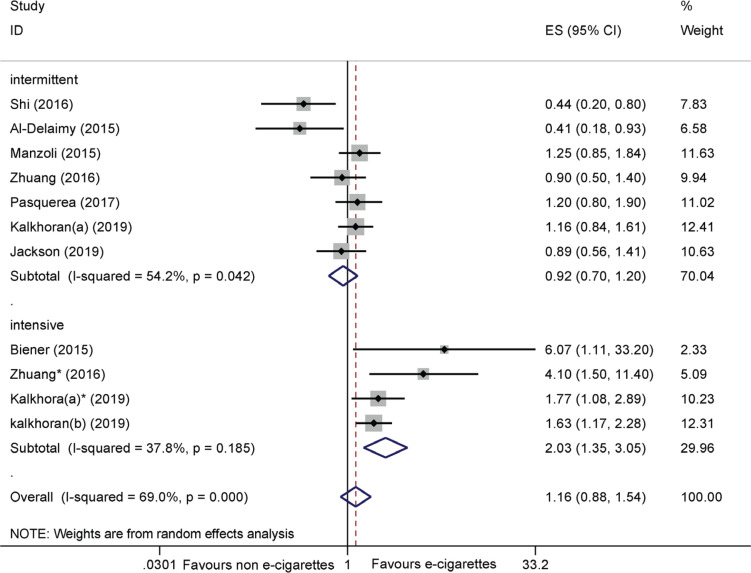
Meta-analysis of cohort studies comparing ever e-cigarette use with non-use on smoking cessation

Nine cohort studies^37-45^ reported the adjusted odds ratio (AOR) for cessation. The pooled results suggested that e-cigarettes were not associated with smoking cessation (AOR=1.16; 95% CI: 0.88–1.54; I^2^=69.0%; 9 trials, n=22220) (Figure 4). Subgroup analysis on the frequency of e-cigarette use suggested that intensive e-cigarette use was more effective in achieving cessation than non-use (AOR= 2.03; 95% CI: 1.35–3.05; I^2^=37.8%; 4 trials, n=1144) (Figure 4).

#### Smoking initiation

##### Findings from one systematic review

Only one systematic review^31^ based on cohort studies involving 17389 young people aged 14–30 years was identified. This review indicated that ever e-cigarette users were more likely to initiate cigarette smoking at follow-up than never users (23.2% vs 7.2%) (AOR=3.5; 95% CI: 2.38–5.16; I^2^=56.0%; 7 trials, n=8759).

##### Findings from 15 cohort studies

**Figure S1:**
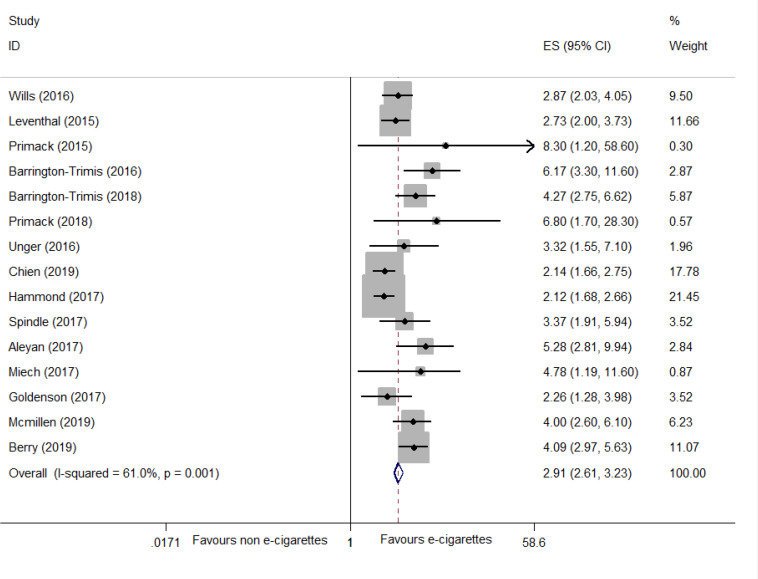
Meta-analysis of cohort studies comparing ever e-cigarette use with non-use on smoking initiation

Fifteen cohort studies^46-60^ were included. The pooled results suggested that ever e-cigarette users were more likely to initiate smoking than non-e-cigarette users (AOR=2.91; 95% CI: 2.61–3.23; I^2^=61.0%; 15 trials, n=68943) (Supplementary file, Figure S1). Subgroup analysis on the frequency of e-cigarette use was not available, since only one trial^52^ reported the AOR of intensive and intermittent e-cigarette use. Sensitivity analysis was also unavailable due to insufficient data. The funnel plot based on the AORs of smoking initiation (Supplementary file, Figure S2) appeared to be asymmetrical, suggesting that there was potential publication bias.

#### Adverse events

Of the 35 included studies, adverse events were reported in 5 studies^27-29,32,34^. Two systematic reviews^27,28^ reported minor adverse short-term events related to e-cigarettes. These included irritation of the mouth and throat, cough and respiratory diseases, but long-term safety is still unknown. Two studies^29,32^ reported that there was no statistical difference in adverse events between the e-cigarette group and control group. One RCT^34^ reported that adverse events were significantly lower in the e-cigarette group (6.7%) compared with the NRT group (17.3%); adverse events included oral pain, cough, headache, and nausea. No serious adverse events were reported in the included trials.

## DISCUSSION

### Main findings

In the light of current policies on e-cigarettes in China and the US, it was timely to conduct a rapid review to comprehensively evaluate the benefits and risks of e-cigarettes. Thirty-five studies published from January 2015 to June 2020 were evaluated and included 6 systematic reviews, 24 cohort studies and 5 RCTs. The study population included adult smokers with or without intention to cease smoking and non-cigarette smoking adolescents. Four systematic reviews^27-30^ indicated that e-cigarettes were superior to placebo for cigarette smoking cessation. Another systematic review^26^ reported that the odds of cessation were 28% lower in the e-cigarette group than the non-use group. Five RCTs (two^33,36^ placebo or no treatment controlled and three^32,34,35^ NRT controlled) reported that the abstinence rate in the e-cigarette group was 2.6% higher than that in the control group, suggesting that e-cigarettes may be more effective than NRT or placebo in achieving smoking cessation among adult smokers. However, this evidence was downgraded due to the small number of events and inconsistencies as assessed using GRADE. Nine cohort studies involving 22220 adult smokers found that e-cigarettes were not superior to non-e-cigarette use for cessation. Subgroup analysis suggested that intensive e-cigarette use (daily or regular use for at least one month) may contribute to cessation, while intermittent or irregular use did not. In light of the limited number of RCTs and the findings from cohort studies on smoking cessation, we could not draw robust conclusions from current evidence. In terms of smoking initiation, one systematic review^31^ reported that ever or past 30-day e-cigarette use was more likely to initiate smoking among teenagers. Updated estimates (AORs) of 15 cohort studies^46-60^ involving 68943 adolescents suggested that ever e-cigarette users were nearly 3 times more likely than non-users to begin smoking cigarettes. However, the evidence was limited due to potential bias (self-reported outcomes, high dropouts and variations in the length of follow-up). No serious adverse events were reported in the included studies.

### Comparisons with other studies

Previous systematic reviews^26-30^ have merely focused on the benefits of e-cigarettes for smoking cessation or the risks for smoking initiation^31^. Former systematic reviews were based on primary studies such as RCTs and cohort studies. We identified the latest systematic reviews, RCTs and cohort studies published in the last six years to provide comprehensive and rapid evidence for policy makers. This is the first rapid review to evaluate the benefits and risks of e-cigarettes, comprehensively. A newly published Cochrane systematic review^61^ found that nicotine e-cigarettes were superior to placebo e-cigarettes or NRT for smoking cessation. This was consistent with the findings from 5 newly conducted RCTs^32-36^ in this rapid review. In addition, this review conducted a subgroup analysis based on the frequency of e-cigarette use.

### Implications

For the study design, future studies should consider the frequency of e-cigarette use, the concentration of nicotine in e-cigarettes and the type of e-cigarettes, since the quitting or initiating smoking effects of e-cigarette use could be potentially affected by those factors. E-cigarette products change very rapidly, especially the Juul type e-cigarettes, which use nicotine salt and can deliver a higher dose of nicotine^62^. Juul is the most popular e-cigarette in the US^63^. Therefore, it is essential to study new products in the future in terms of their effect on quitting or initiating cigarettes. The definition of smoking cessation or smoking initiation needs to be clarified, the current standards suggest that the duration is for at least 30 days cessation or smoking initiation at follow-up. Likewise, the effects of electronic cigarettes on smoking cessation among smokers with long-term or short-term smoking may vary. Hence, different smoking durations among smokers should be well reported and stratified in future studies. In terms of outcomes, objective measurements of smoking initiation rather than self-reported measurements are warranted. Cotinine as a biomarker^64^ of smoke exposure can be used to predict smoking initiation among adolescents. Additionally, dropouts should be minimized as much as possible in future cohort studies. Last but not least, the long-term safety of e-cigarette use should also be a future focus. For policy makers, appropriate e-cigarette use may be potentially effective in smoking cessation for adult smokers. However, e-cigarette use in adolescents was potentially associated with subsequent smoking initiation, and therefore the sale of e-cigarettes to minors should be banned completely worldwide.

### Strengths and limitations

Rapid review methodology was employed to provide timely evidence on e-cigarette use due to the urgent need to inform ongoing government policies. This is the first rapid review to evaluate the benefits and risks of e-cigarettes, comprehensively. The latest eligible systematic reviews, RCTs and cohort studies were all identified in order to provide a comprehensive evaluation on e-cigarette use. However, there are several limitations. First, similar to other rapid reviews, the search time was limited from January 2015 to June 2020, and some evidence may have been missed. The results for smoking initiation were at risk of publication bias. Second, though the evidence from 5 RCTs suggested that e-cigarettes were superior to NRT or placebo for smoking cessation, the certainty of evidence was downgraded due to imprecision and inconsistency. Additionally, evidence from 9 cohort studies suggested that ever e-cigarette use was not associated with smoking cessation. Thus, the smoking cessation effect of e-cigarettes still could not be determined based on current evidence.

## CONCLUSIONS

Low certainty evidence suggests that e-cigarettes appear to be potentially effective in smoking cessation for adult smokers. However, this beneficial effect needs to be further confirmed in large sample, well designed and fully reported trials. The use of e-cigarettes in adolescents may be associated with subsequent smoking initiation. No serious adverse events were reported in the included studies, however, the long-term safety of e-cigarettes should also be a future focus.
